# 8-OH-DPAT (5-HT1A agonist) Attenuates 6-Hydroxy- dopamine-induced catalepsy and Modulates Inflammatory Cytokines in Rats

**Published:** 2013-12

**Authors:** Hamdolah Sharifi, Alireza Mohajjel Nayebi, Safar Farajnia

**Affiliations:** 1 Drug Applied Research Center, Tabriz University of Medical Sciences, Tabriz, Iran; 2 Department of Pharmacology and Toxicology, Faculty of Pharmacy, Tabriz University of Medical Sciences, Tabriz, Iran; 3 Biotechnology Research Center, Tabriz University of Medical Sciences, Tabriz, Iran

**Keywords:** 8-OH-DPAT, Catalepsy, Chronic, Cytokines, Rat

## Abstract

***Objective(s):*** Neuroinflammation in Parkinson disease (PD) is associated with glial cells activation and production of different inflammatory cytokines. In this study, we investigated the effect of chronic administration of 8-OH-DPAT on 6-OHDA-induced catalepsy and levels of inflammatory cytokines in cerebrospinal fluid (CSF).

***Materials and Methods:*** Catalepsy was induced by unilateral infusion of 6-OHDA (8 μg/2 μl/rat) into the central region of the sabstantia nigra pars compacta (SNc) being assessed by the bar-test, 5, 60, 120 and 180 min after intraperitoneal (IP) administration of 8-OH-DPAT (5-HT_1A _receptor agonist; 0.25, 0.5 and 1mg/kg, IP for 10 days). CSF samples were collected on the tenth day of 8-OH-DPAT administration and analyzed by ELISA method to measure levels of TNF-α, IL-1β and IL-6.

***Results:*** Chronic injection of 8-OH-DPAT decreased catalepsy in a dose dependent manner when compared with the control group. The most anti-cataleptic effect was observed at the dose of 1 mg/kg of 8-OH-DPAT. Levels of TNF-α in CSF increased three weeks after 6-OHDA injection while there was a significant decrease in TNF-α level of parkinsonian animals treated with 8-OH-DPAT (1 mg/kg, IP for 10 days). IL-1β and IL-6 decreased and increased in parkinsonian rats and in 8-OH-DPAT-treated parkinsonian rats, respectively.

***Conclusion:*** Our study indicated that chronic administration of 8-OH-DPAT improves catalepsy in 6-OHDA-induced animal model of PD and restores central concentration of inflammatory cytokines to the basal levels. 5-HT_1A _receptor agonists can be suggested as potential adjuvant therapy in PD by modulation of cerebral inflammatory cytokines.

## Introduction

Parkinson disease (PD) is the second most common and progressive neurodegenerative disease caused mainly by loss of dopaminergic neurons in the substantia nigra pars compacta (SNc). The major movement symptoms are rigidity, akinesia, tremor and postural abnormalities as well as cognitive disturbances (1).

The role of neuroinflammation in degeneration of nigrostriatal neurons is of interest to many investigators (2, 3). The first evidence for the role of inflammation in PD came from an observation by McGeer and colleagues on activated microglia and T cells in the post-mortem SNc of a patient suffering from PD (4). Epidemiological studies have shown that the incidence of idiopathic PD is lower in chronic users of anti-inflammatory drugs (5, 6). Neuroinflammation is regulated by many signal molecules including cytokines. They are multifuncti-

onal proteins and in the CNS, play a role in the normal development of the brain as well as in neuro-immuno-pathological processes following injury and neurodegeneration (7). Several studies have reported significant increase of pro-inflammatory cytokines such as IFN-γ, IL-1β and TNF-α, being expressed by glial cells in the nigrostratial regions of patients with PD (4, 8-10). In general, pro-inflammatory cytokines such as TNF-α has neurotoxic effects, while IL-6 and IL-1β, classical pro-inflammatory cytokines, have a dual effect. For instance, low concentrations of IL-6 protect neuronal cells from death, while larger concentrations are neurotoxic (11, 12). 

Previous studies have shown that serotonergic system is involved in PD (13-15). Serotonergic projections originating from the dorsal raphe nuclei innervate all parts of the basal ganglia and play a role in the regulation of movements executed by the basal ganglia (14). In this context, role of 5-HT_1A_ receptors in motor impairments of PD is the center for attention (13, 16). Activation of these receptors could decrease serotonin release and subsequently improve motor function in 6-OHDA-lesioned rats (17, 18). Studies have shown that stimulation of the 5-HT_1A_ receptor attenuates anoxia-induced apoptosis in the neuronal HN2-5 (Hippocampal neuron-derived cell line) cells (19). It seems that cytokines act as messengers between the immune system and the brain, exerting their effect on serotonergic system through several processes such as degradation of their precursor, tryptophan (20). The role of pro-inflammatory cytokines in the pathogenesis of PD has been shown in several studies (12, 21, 22), although the effect of chronic administration of 5-HT_1A_ receptor agonists on 6-OHDA-induced catalepsy and the role of cytokines such as TNF-α, IL-1β and IL-6 has not been clearly studied yet. Thus, in this study we attempted to investigate the effect of chronic administration of 8-OH-DPAT on 6-OHDA-induced catalepsy and possible involvement of TNF-α, IL-1β and IL-6. 

## Materials and Methods

***Chemicals***

All chemicals were obtained from Sigma Chemical Co. (USA), except for ELISA kits, which were purchased from eBioscience Co. (Austria). All solutions were prepared freshly on the experimentation day. 8-OH-DPAT (a 5-HT_1A_ receptor agonist) and 6-OHDA were dissolved in physiological saline (0.9% NaCl) and 0.9% saline containing 0.2% (w/v) ascorbic acid, respectively. 6-OHDA was injected into the central region of the substantia nigra pars compacta (SNc) in a total volume of 2 μl /rat with a constant injection rate of 0.2 μl /min.

***Animals***

The experiments were carried out on male Wistar rats weighing 270-300 g. Animals were housed in standard polypropylene cages, four per cage, under a 12:12 hr light/dark schedule at an ambient temperature of 25 ± 2°C and were allowed food and water *ad libitum*. Animals were acclimated to the testing conditions for 2 days before the behavioral experiment was conducted. All procedures were carried out under the ethical guidelines of the Tabriz University of Medical Sciences. Twenty-four rats were divided into three groups: normal, sham-operated (receiving 2 μl vehicle) and 6-OHDA (8 μg/2 μl/rat)-injected.

***6-OHDA-induced SNc lesion***

Animals were anesthetized with an IP injection of ketamine (50 mg/kg) and xylazine (5 mg/kg). After being deeply anesthetized (loss of corneal and toe pad reflexes), rats were mounted in a Stoelting stereotaxic frame in the flat skull position. The scalp was shaved, swabbed with povidone-iodine 10%, and a central incision made to expose the skull. 6-OHDA was injected thorough a guide cannula (23 gauge stainless steel) implanted in the SNc. The coordinates for this site were based on the rat brain atlas (23) as follows: anteroposterior (AP): –5.0 mm from the bregma; mediolateral (ML): –2.1 mm from the midline and dorsoventral (DV): –7.7 from the skull. 

Desipramine (25 mg/kg, IP) was injected 30 min before the intra-SNc injection of 6-OHDA to avoid destruction of noradrenergic neurons. Thereafter, 6-OHDA (8 μg per rat in 2 μl saline with 0.2% ascorbic acid) was infused with an infusion pump at a constant flow rate of 0.2 μl/min into the left SNc. At the end of the infusion, the injection tube was kept implanted for an additional 2 min and then was slowly retracted. Sham-operated animals were submitted to the same procedure but 2 μl vehicle (0.9% saline containing 0.2% (w/v) ascorbic acid) instead of 6-OHDA was infused into the SNc.

***Catalepsy test***

Catalepsy was measured using a standard bar test 21 days after 6-OHDA and 10 days after IP injection of 8-OH-DPAT. In this method, forepaws of rats were placed over a 9-cm-high standard wooden bar, and the duration of retention of rats in this imposed posture was considered as the bar test elapsed time. The end point of catalepsy was considered when both front paws were removed from the bar or when the animal moved its head in an exploratory manner. The cut-off time of the test was 600 sec. The test was carried out 5, 60, 120 and 180 min after drug administration on the 10^th^ day. All observations were made between 9 am and 4 pm. After a three-week recovery period, only the rats being markedly immobilized in the bar test were subjected to further experimentation (parkinsonian rats). Afterwards, the parkinsonian rats were randomly divided into equal groups and received IP injections of 8-OHD-PAT (0.25, 0.5 and1 mg/kg, IP) once daily (9 a.m.) for 10 days. 

***CSF sampling***

CSF samples were collected on day 10 of 8-OH-DPAT administration. Animals were anesthetized by IP injections of ketamine (50 mg/kg) and xylazine (5 mg/kg) and mounted in a Stoelting stereotaxic frame. The skull was kept in 45 ° position and CSF was aspirated using a sterile 100 µl syringe 23gauge needle. The CSF samples were kept at -70°C until being assessed by Enzyme-linked immunosorbent assays (ELISA) method.

***Analysis of TNF-α, IL-1β and IL-6 expression by ELISA***

 ELISA method was employed for determination of TNF-α, IL-1β and IL-6 in CSF samples. Assays were performed by a commercial ELISA kit (IBL INTERN-ATIONAL GMBH) as per manufacturer's instructions and in the similar conditions for all assays. Briefly, the frozen CSF samples were diluted, added into the wells and incubated at room temperature for 120 min on a microplate shaker. Subsequent to washing, diluted Streptavidin-Horseradish peroxidase-conjug-ated antimouse TNF A, IL-1B and IL-6 were reacted for 60 min at room temperature (on microplate shaker set at 200 rpm). After washing for second time, the wells were developed with tetramethyl benzidine (TMB) for 10 min and the optical densities were read at 450 nm with an ELISA reader. 

**Figure 1:**
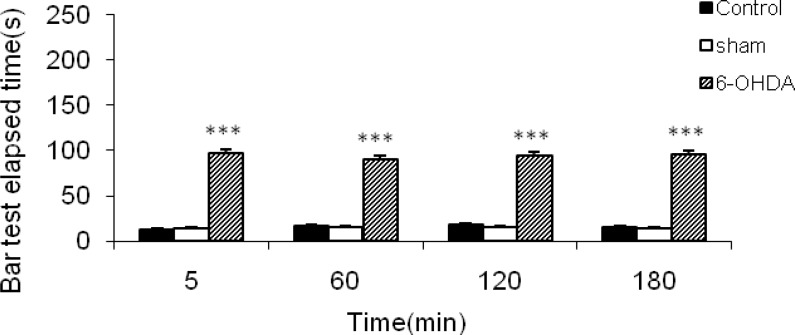
The results of bar test in control, sham-operated and 6-OHDA (8 μg/2 μl/rat)-lesioned rats. Each bar represents the mean ± SEM of bar test elapsed time (sec); n = 8 rats in each group; *** *p* <0.001 when compared with normal and sham-operated groups

***Histology***

All animals having guide cannula were sacrificed at the end of the experiments. Brain dissections were performed in all animals to confirm the exact implantation of guide cannula into the SNc. Brain in the injecting tube in situ was fixed in 10% formalin for 1 week. The location of the tip of the injecting tube was then verified in serial sections. Only the results from bar tests in animals with the tip of the injecting tube within the SNc area were used for statistical analysis.

***Statistical analysis***

Statistical analysis for each data set was calculated by SPSS software (version 16.0). Data were expressed as mean+SEM, and one-way ANOVA test was utilized to analyze in the data from behavioral and biochemical experiments. In the case of significant variation (*P*< 0.05), the values were compared by Tukey test.

## Results

***6-OHDA-induced catalepsy***

6-OHDA was able to induce significant (*P* < 0.001) catalepsy in comparison with both normal and sham-operated rats (Figure 1).

***Effect of 8-OH-DPAT on 6-OHDA-induced catalepsy***

**Figure 2:**
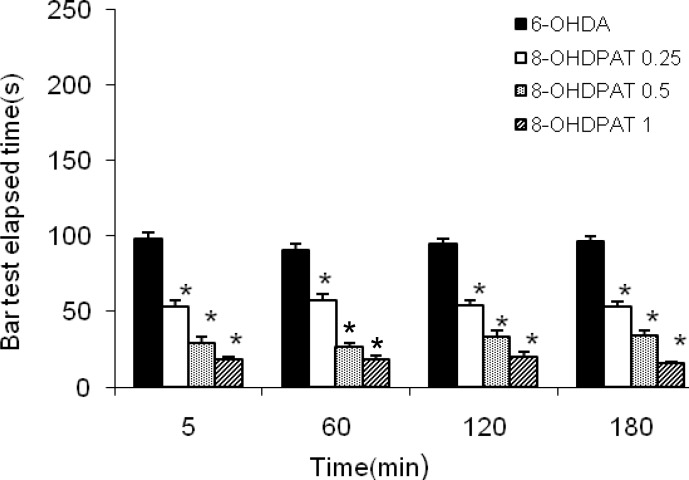
The results of bar test in 6-OHDA (8 μg/2 μl/rat)-lesioned rats treated with 8-OH-DPAT (0.25, 0.5, and1 mg/kg, IP for 10 days). Each bar represents the mean± SEM of catalepsy time (sec); n = 8 rats in each group; * *p* < 0.001 when compared with 6-OHDA-lesioned rats

Four groups of 6-OHDA-lesioned rats received saline or one of the three different doses of 8-OH-DPAT (0.25, 0.5 and1 mg/kg, IP), respectively for 10 days. The results showed that 8-OH-DPAT attenuated the severity of 6-OHDA-induced catalepsy (*P*< 0.001) (Figure 2).

**Figure 3:**
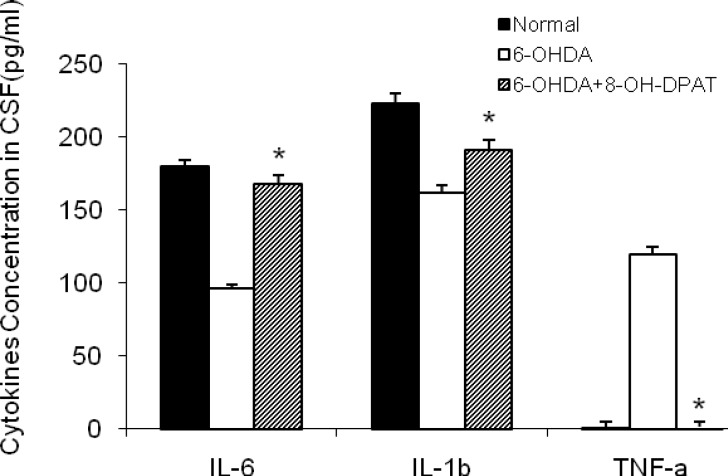
The CSF concentration of cytokines (IL-6, IL-1β and TNF-α) in the control, 6-OHDA-lesioned rats and 6-OHDA -lesioned rats treated with 8-OH-DPAT (1 mg/kg, IP for 10 days). Each bar represents the mean± SEM of cytokines concentration in the CSF (pg/ml) n = 8 rats in each group; * *P*< 0.001 when compared with 6-OHDA-lesioned rats

***Effect of 8-OH-DPAT on inflammatory parameters***

Results showed that TNF-α was undetectable in normal rats while it was increased three weeks after 6-OHDA injection and decreased following 10 days of IP administeration of 8-OH-DPAT to normal animals. Levels of the two other cytokines were decreased in 6-OHDA-lesioned rats in comparison with normal group but increased 10 days after injection of 8-OH-DPAT (Figure 3).

## Discussion

In our previous studies (13, 24) the potential anticataleptic effects of 5-HT_1A_ receptor agonists in 6-OHDA-lesioned rats has been investigated in single dose administrations. Given the chronic clinical administrations of such drugs, we investigated the potential anticataleptic effects of chronic administration of 8-OH-DPAT in 6-OHDA-lesioned rats in the current study. Our results showed that intra-SNc injection of 6-OHDA induced catalepsy in animals when assessed by the bar test (13). Bar test is a standard test being frequently used for evaluation of catalepsy induced by 6-OHDA and neuroleptic drugs in rodents (25). According to the results, chronic administration of 8-OH-DPAT, an agonist of 5-HT_1A_ receptors, improved catalepsy in 6-OHDA-lesioned rats in a dose dependent manner. Such finding confirms the previous studies, reporting a promising role for 5-HT_1A _agonists in decreasing the motor disorders associated with PD (24). 

5-HT_1A_ receptors are widely distributed throughout the basal ganglia. They are located on dorsal raphe neurons with efferents to the striatum and on cortical neurons that send glutamatergic projections into the basal ganglia (26). In the basal ganglia, serotonin modulates dopamine-related motor activity through affecting the 5-HT_1A_ receptor (27). It has been shown that 5-HT_1A_ agonists improve motor impairments in parkinsonian animals via stimulation of somatodendritic 5-HT_1A_ receptors and subsequent decrease in serotonin release from the nerve endings (28). Furthermore, studies have indicated that 5-HT_1A_ receptor plays a role in neuronal survival (29, 30) and has a neuroprotective effect in animal models of stroke and traumatic brain injury (29, 31). Such effect is exerted through inhibition of glutamate release that leads to a reduction in the putative excitotoxicity-mediated cell death (31). 

Herein, we assessed levels of inflammatory cytokines i.e TNF-α, IL-6 and IL-1β in the CSF of parkinsonian rats being treated with chronic injections of 8-OH-DPAT. According to our results, there was a significant increase in the amount of TNF-α in parkinsonian rats whereas its levels were resorted to normal ranges by chronic administration of 8-OH-DPAT. This is in accordance with other studies reporting that toxic effects of 6-OHDA are in part mediated through the activation of microglia and increasing levels of TNF-α in both SN and striatum (4, 9, 32 ). The substantia nigra (SN) has high density of microglia and it is hypothesized that DA neurons are susceptible to inflammatory damage as a major stimuli for neurodegenerative diseases (33). Activated microglia release proinflammatory cytokines such as TNF-α that play a key role in modulation of inflammatory responses (34).

The levels of IL-1β and IL-6 were decreased in parkinsonian rats when compared with normal (non-parkinsonian) animals. In parkinsonian rats, which were treated with chronic injections of 8-OH-DPAT, the levels of IL-1β and IL-6 were increased to that of normal rats. It has been reported that there is an increase in CSF concentration of IL-1β and IL-6 in parkinsonian rats (32, 35). In addition to their pro-inflammatory effect, these are pleiotropic cytokines, which can produce neuroprotective effects in PD, Alzheimer disease (AD) and CNS injuries (32, 36). Our results showed that IL-1β and IL-6 were decreased in 6-OHDA-lesioned rats while in 6-OHDA-lesioned rats, being treated with 8-OH-DPAT, the levels of IL-1β and IL-6 were restored to normal values. This is in agreement with previous studies which suggest a neuroprotective effect for these cytokines (22, 37).

## Conclusion

Our data suggest that chronic administration of 8-OH-DPAT improves catalepsy in 6-OHDA-lesioned rats. Moreover, we suggest that 5-HT_1A_ receptor agonists can be utilized as adjuvant therapy along with commonly used anti-parkinsonian drugs. However, further clinical investigations should be carried out to prove this. 
